# State-Independent and -Dependent Structural Connectivity Alterations in Depression

**DOI:** 10.3389/fpsyt.2020.568717

**Published:** 2020-11-30

**Authors:** Yiming Fan, Jin Liu, Ling-Li Zeng, Qiangli Dong, Jianpo Su, Limin Peng, Hui Shen, Xiaowen Lu, Jinrong Sun, Liang Zhang, Mi Wang, Jugessur Raj, Bangshan Liu, Dewen Hu, Lingjiang Li

**Affiliations:** ^1^College of Intelligence Science and Technology, National University of Defense Technology, Changsha, China; ^2^Department of Psychiatry, The Second Xiangya Hospital, Central South University, Changsha, China; ^3^Hunan Key Laboratory of Psychiatry and Mental Health, China National Clinical Research Center on Mental Disorders (Xiangya), China National Technology Institute on Mental Disorders, Hunan Technology Institute of Psychiatry, Mental Health Institute of Central South University, Changsha, China

**Keywords:** major depressive disorder, structural connectivity, state-independent, state-dependent, DTI

## Abstract

Some brain abnormalities persist at the remission phase, that is, the state-independent abnormalities, which may be one of the reasons for the high recurrence of major depressive disorder (MDD). Hence, it is of great significance to identify state-independent abnormalities of MDD through longitudinal investigation. Ninety-nine MDD patients and 118 healthy controls (HCs) received diffusion tensor imaging scanning at baseline. After 6-month antidepressant treatment, 68 patients received a second scan, among which 59 patients achieved full clinical remission. Differences in whole-brain structural connectivity (SC) between patients with MDD at baseline and HCs were estimated by two-sample *t*-tests. Masked with significantly changed SCs in MDD, two-sample *t*-tests were conducted between the remitted MDD subgroup at follow-up and HCs, and paired *t*-tests were implemented to compare the differences of SC in the remitted MDD subgroup before and after treatment. Significantly decreased SC between the right insula and the anterior temporal cortex (ATC), between the right ATC and the posterior temporal cortex (PTC), between the left ATC and the auditory cortex as well as increased connectivity between the right posterior cingulate cortex (PCC) and the left medial parietal cortex (MPC) were observed in the MDD group compared with the HC group at baseline (*p* < 0.05, FDR corrected). The decreased connectivity between the right insula and the ATC and increased connectivity between the right PCC and the left MPC persisted in the remitted MDD subgroup at follow-up (*p* < 0.05, FDR corrected). The decreased SC between the right insula and the ATC and increased SC between the right PCC and left MPC showed state-independent characters, which may be implicated in the sustained negative attention bias and motor retardation in MDD. In contrast, the decreased SC between the right ATC and the PTC and between the left ATC and the auditory cortex seemed to be state-dependent.

## Introduction

Major depressive disorder (MDD) is characterized by a high rate of recurrence and a high rate of lifetime prevalence, which brings an enormous burden to the patients, families, health system and society (1). Many patients with MDD have residual depressive symptomatology at remission, which seems to be a significant predictor of relapse (2). MDD residues include persistent subclinical illness symptoms and potential persistent brain abnormalities. Therefore, identification of state-independent biomarkers may provide insights into MDD pathophysiology and pathogenesis.

Whole-brain white-matter structural connectivity (SC), derived from diffusion tensor imaging (DTI) tractography, are the substrate of distributed functional interactions among brain regions. Tymofiyeva et al. compared the DTI-based structural networks in a cohort of 57 depressed adolescents and 41 matched healthy controls (HCs) and found that MDD patients showed reduced SC between the insula and the right caudate (3). Korgaonkar et al. used the inter-regional SC of the entire cortex to characterized MDD. They found that the most discriminant features included the SC of the right insula to the right inferior parietal (4). Furthermore, Qin et al. explored the topological properties of structural brain networks of MDD and found that both current and remitted patients exhibited a decrease in node strength of the right insula compared with HCs (5). Another investigation in patients with remitted geriatric MDD revealed an altered component including 18 regions and 19 SCs in the right hemisphere. Compared with the HCs, all the connectivity in the component was decreased in the patients. The regions were mainly paralimbic (insula, parahippocampal gyrus, and superior/middle temporal gyrus), subcortical (hippocampus, caudate nucleus, putamen, pallidum, and thalamus) regions (6). Taken these findings together, abnormal SC with insula in MDD patients seems to show state-independent characters. In addition, many previous studies had found decreased inter-regional SC in the temporal-limbic, frontolimbic, and parietal-limbic circuits in MDD patients relative to HCs (4, 7–9). Recently, reduced connections related to the superior temporal gyrus, both for functional and structural connectivity, were also found in MDD patients relative to HCs (10). After electroconvulsive therapy, the decreased SCs among the temporal, frontal lobe, and limbic structures in MDD patients were reversed (11). Additionally, increased communication between the left superior temporal gyrus and the right precuneus was reported in remitted MDD relative to current MDD (12). In this way, abnormal SC with the temporal lobes seems to show a state-dependent character. These previous studies revealed the state-independent and -dependent SC in MDD to some extent. However, these conclusions are inconvergent, and most of them are based on cross-sectional studies. Only a few prospective follow-up studies examined the course of SC changes in MDD with a 2-month antidepressant treatment or a short-time electroconvulsive therapy (5, 11). Besides, these few studies had a small sample size and showed a lack of HCs or a lack of controlling medication. Therefore, large-scale follow-up studies with a more extended period are needed to overcome these shortcomings.

To pinpoint the state-independent and -dependent SC abnormalities in MDD patients clearly, a group of MDD patients without medication for at least 2 weeks at baseline were recruited in this study. Patients were treated with paroxetine during the 6-month follow-up, and they received a DTI scan respectively at baseline and the sixth-month follow-up. Drawing on the findings of former studies, we hypothesized that the insula-related SC abnormalities may be state-independent, and the temporal-related SC abnormalities may be state-dependent.

## Methods and Materials

### Subjects and Design

A total of 107 depressed patients were recruited at the outpatient or inpatient departments of Zhumadian Psychiatric Hospital, Henan province, China. Both MDD patients and 123 demographically matched HCs underwent the MRI scan at baseline. The frequent inclusion criteria for the MDD group and the HC group were: (1) right-handed; (2) 18–55 years old; (3) education ≥6 years; Additional inclusion criteria for the MDD group were: (1) diagnosis of a current major depressive episode by an attending psychiatrist using the Structured Clinical Interview for Diagnostic and Statistical Manual of Mental Disorders-IV (SCID); (2) scored more than 20 on the Hamilton Depression Rating Scale (HAM-D_24_); (3) no psychotropic drugs for at least 2 weeks (fluoxetine for 6 weeks) before inclusion. Inclusion criteria for HC group: (1) scored less than eight on the HAM-D_24_; (2) no history of mental disorders. Exclusion criteria for both groups included a history of severe somatic diseases, pregnant or breast breeding women, having used medications for thyroid diseases, glucocorticoids, or anticoagulants (heparin, warfarin, etc.) in the past 3 months, abnormal urine toxicology or thyroid screening results, and current or past alcohol or substance abuse or dependence. Subjects with psychiatric diagnoses other than depression were excluded.

**Figure 1:**
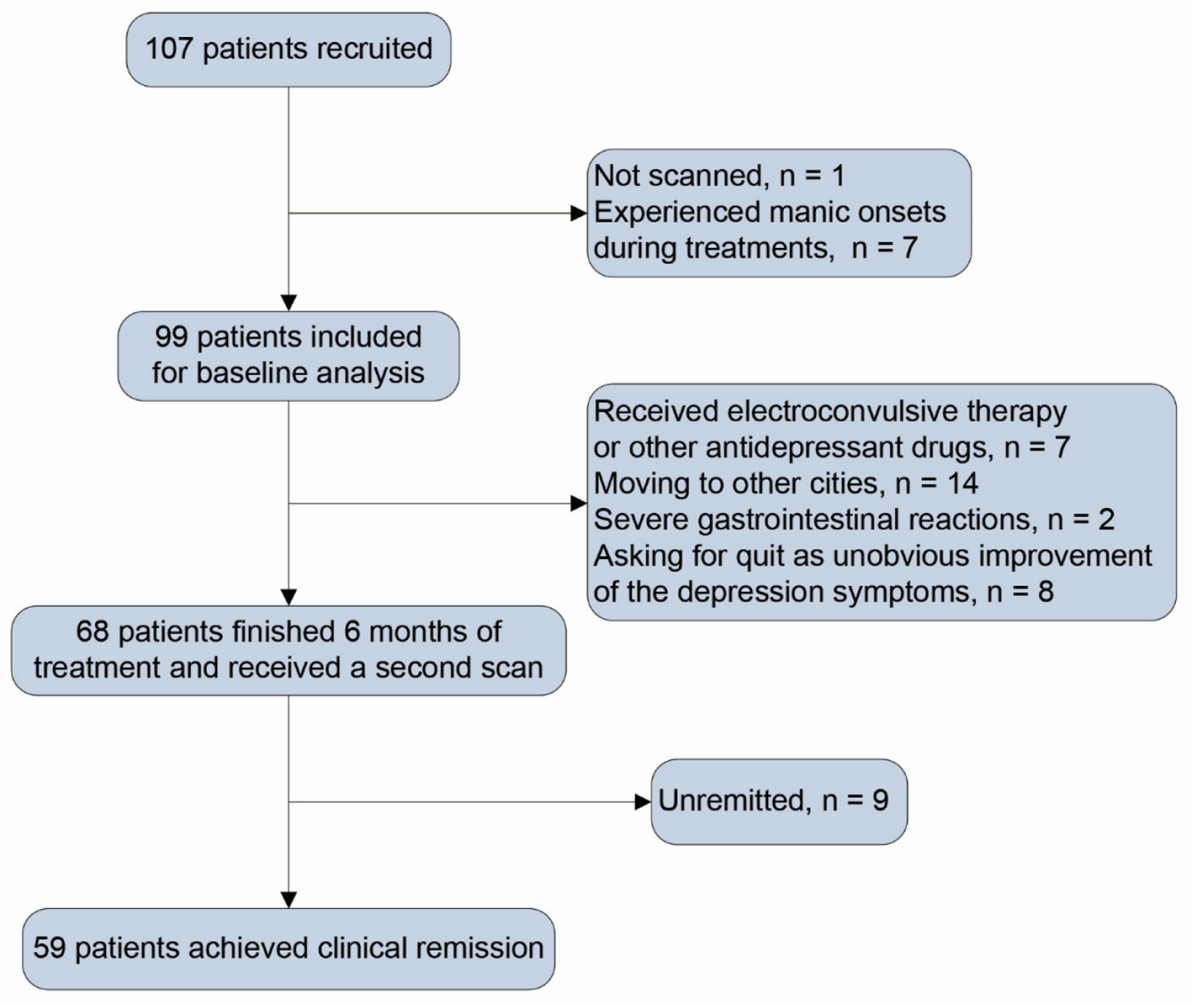
Flow chart.

After baseline assessment, patients received a 6-month paroxetine treatment. In the first week, patients received paroxetine 10 mg/day (d), the minimum dose for the study. In the second week, patients received paroxetine 20 mg/d or higher doses depending on clinical symptoms, side effects, and clinical antidepressant effects. The maximum dose of paroxetine was 60 mg/d. Depressive symptoms were assessed with the HAM-D_24_ per month for the next 6 months. Patients received other clinical assessments and a second scan at the endpoint of the sixth month. Of the initial 107 patients, one was not scanned, and seven experienced manic onsets in the follow-up period. In consequence, 99 patients were included for baseline analysis. During follow-up, seven patients received other antidepressant medications or electroconvulsive therapy, 24 patients did not continue to participate as some uncontrollable factors, and 68 patients finished 6-month treatment and underwent a second scan. A flow chart was shown in Figure 1 to introduce the patients' information in detail after inclusion, exclusion, medications, and MRI scanning. Patients who scored less than eight on HAM-D_24_ for at least two consecutive months and persisted till the end of the 6 months of follow-up were examined clinically remitted. After treatment, 59 patients were considered clinically remitted (remitted MDD subgroup), and nine failed to achieve clinical remission. Due to the small sample size of the non-remitters, there was a lack of sufficient powers while comparing the non-remitters with other groups. We thus focused on analyzing the SC changes in remitted MDD subgroup.

This longitudinal investigation was approved by the Ethics Committee of Zhumadian Psychiatric Hospital and the Ethics Committee the Second Xiangya Hospital of Central South University, and written informed consent was obtained from all subjects.

### Image Acquisition and Preprocessing

DTI data were collected using a 3T Signa HDxt scanner. Diffusion tensor images were acquired adopting a single-shot echo-planar imaging sequence (EPI), 32 non-collinear directions (b = 1,000 s/mm^2^), one non-diffusion weighted volume (b = 0 s/mm^2^), TR = 13,000 ms; slice thickness = 3 mm, FOV = 256 × 256 mm, 128 × 128 matrix, NEX = 1, and 50 gap-free transverse slices covering the whole brain. We also obtained high-resolution 3D structural images using a T1-weighted BRAVO sequence, TR = 6.8 ms, slice thickness = 1 mm, slice gap = 0 mm, flip angle = 9°, TE = 2.5 ms, turnover time (TI) = 1,100 ms, FOV = 256 × 256 mm, 256 × 256 matrix, NEX = 1. The 3D brain image of each subject contains 192 consecutive sagittal slices.

All DTI data were processed with the PANDA package based on FSL (FMRIB's Software Library, http://www.fmrib.ox.ac.uk/fsl) (13).

The DTI data were preprocessed by the following four steps: (1) eddy current and motion artifact correction; (2) diffusion tensor estimation, (3) fractional anisotropy (FA) calculation, and (4) diffusion tensor tractography. The eddy current distortions and motion artifacts were corrected by applying an affine alignment of each diffusion-weighted image to the non-diffusion weighted image. After this step was completed, the DTI elements were evaluated by solving the Stejskal and Tanner equation. Next, three eigenvalues and eigenvectors were acquired by diagonalizing the reconstructed tensor matrix. And then according to the three eigenvalues, the FA value of each voxel was calculated. The tractography was performed using the “fiber assignment by continuous tracking (FACT)” method (14) in the last step. All the tracts in the dataset were calculated by seeding per voxel with a FA ≥ 0.2. The tractography was terminated if it turned an angle ≤ 45° or reached a voxel with a FA ≤ 0.2 (14). The tractography was implemented in each participator to generate three-dimensional curves representing the connectivity of fiber bundle (15, 16). Tens of thousands of streamlines were drawn, etching out the main white matter fiber tracts.

### SC Network Constructions

#### Network Nodes Definition

The procedure for defining nodes has been formerly described (17). Here, we adopted a functional parcellation of the human cerebral cortex and striatum (18, 19), which had been used in previous studies (20–22). The parcellation divided the cerebral cortex and divided striatum into 132 regions totally according to the 17-functional network parcellation of the human brain (not including the cerebellar regions). These regions were applied to represent nodes in the SC networks. The list of these brain regions of the SC network was shown in Supplementary Table 1. The original parcellation was in the Montreal Neurological Institute (MNI) space. To establish the nodes of the SC network in each participant, regions should be defined in the native diffusion space (23). In short, the T1-weighted images of a subject were coregistered to the b0 image in the native diffusion space. And the converted T1 images were converted into the ICBM152 T1 image in the MNI space nonlinearly. And then the inverse transformations were applied to the above parcellation. In this way, we obtained subject-specific parcellation of the brain, each region representing a node in the network in the native diffusion space.

#### SC Definition

To define the network edge across each pair of the 132 regions, the average FA value of all voxels connected by tracts between each pair of areas was calculated. We set the average FA value of the connected fibers between two areas as the weight of the network edge. To reduce the potential impact of data acquisition and pre-/post-processing on noise or other factors during diffusion tractography, we took two regions as structurally connected if the number of streamlines between two regions was >3 (17, 24). The threshold selection decreased the risk of false-positive connectivity owing to the limitations or noise in the tractography. Consequently, a FA-weighted SC network for each subject was constructed, a sparse and symmetric 132 × 132 matrix.

### Statistical Analyses

One sample *t*-test was performed to extract SCs that met a significant level in HCs and MDD patients at baseline. The identified connectivity within either MDD patients or HCs were selected as connectivity of interest (COI) in the following two-sample *t*-tests.

For each connectivity in the COI, a two-sample *t*-test was conducted to explore significantly different connectivity between MDD patients at baseline and HCs. With the abnormal SCs in MDD as masks, two-sample *t*-tests were accomplished between the remitted MDD subgroup at follow-up and HCs. And paired *t*-tests were implemented to compare the differences in the remitted and unremitted MDD subgroup before and after treatment. Two-sample *t*-tests were accomplished between the patients who finished the 6-month follow-up procedure and the drop-outs at baseline. The abnormal connectivity was shown with the Surf Ice tool (https://www.nitrc.org/projects/surfice/).

Pearson correlation analyses were conducted to explore the relationship between HAM-D_24_ scores and the abnormal SCs in the MDD group at baseline.

### Validation Analyses

To validate the results with different parcellation schemes, we re-analyzed the data using nodes defined in the Automated Anatomical Labeling (AAL) parcellation. The AAL parcellation (25) has been widely applied to assess SC alteration in MDD (26). The FA-weighted values between each pair of regions were defined as the edges.

To validate the results with different definitions of the network edge, we re-analyzed the data by defining the normalized number of fibers as the weight of the network edge. As the number of fibers tracts between regions of interest (ROI) *i* and ROI *j* of each individual (*N*_i,j_) was obtained from the native diffusion space, the number of fibers were normalized by the sum of surfaces of ROI *i* and ROI *j*, that is 2*N*_i,j_/(*S*_i_ + *S*_j_) as the weight of each edge (27). *S*_*i*_ and *S*_*j*_ are two-dimension intersect of the individual's white matter with the parcellation ROI_*i*_ and ROI_*j*_, respectively.

## Results

### Demographic and Clinical Details of the MDD Group and HC Group

The demographic and clinical details of the MDD group at baseline, HC group, and remitted MDD subgroup at follow-up were shown in Table 1. Demographic, clinical measures were presented as mean ± SD. The MDD group at baseline and the HC group did not significantly differ on age, gender, and years of education. The remitted MDD subgroup and HC group also did not significantly differ on age, gender, and years of education.

**Table 1:** Demographic and clinical characteristics by groups.

**Characteristics**	**BL (Mean ± SD)**	**HC (Mean ± SD)**	**rFU (Mean ± SD)**	**BL vs. HC**	**rFU vs. HC**
^a^Age (years)	34.17 ± 8.79	35.01 ± 8.86	35.42 ± 9.15	*p* = 0.49	*p* = 0.77
^b^Gender (F/M)	57/42	65/58	35/24	*p* = 0.71	*p* = 0.59
^a^Education (years)	10.54 ± 3.40	10.65 ± 3.25	10.78 ± 3.48	*p* = 0.80	*p* = 0.81
Onset age	32 ± 9.08	–	33 ± 8.59	–	–
Total illness length (months)	42 ± 52.30	–	51 ± 60.95		
^a^HAM-D_24_	31 ± 7.55	1.66 ± 1.94	2.42 ± 2.39	*p* < 0.001	*p* = 0.061
^a^HAMA	19.03 ± 6.67	–	2 ± 2		

*BL, MDD patients at baseline; rFU; remitted MDD subgroup at follow-up; HC, healthy control group. vs., versus; SD, standard deviation. HAMA, Hamilton anxiety scale*.

a *p-values were acquired by two-sample t-tests*.

b *p-values were acquired by chi-square tests*.

### SC Abnormalities in MDD Patients at Baseline

Three hundred and fifty-four connectivity in HCs and 378 connectivity in patients at baseline survived in one-sample *t*-tests (*p* < 0.05, FDR corrected). The average number of fibers tracts between different nodes of each survived connectivity in HCs and MDD patients at baseline was shown in Supplementary Figure 1. COI was then defined as the union of these connectivities, which consist of 378 connectivity.

**Figure 2:**
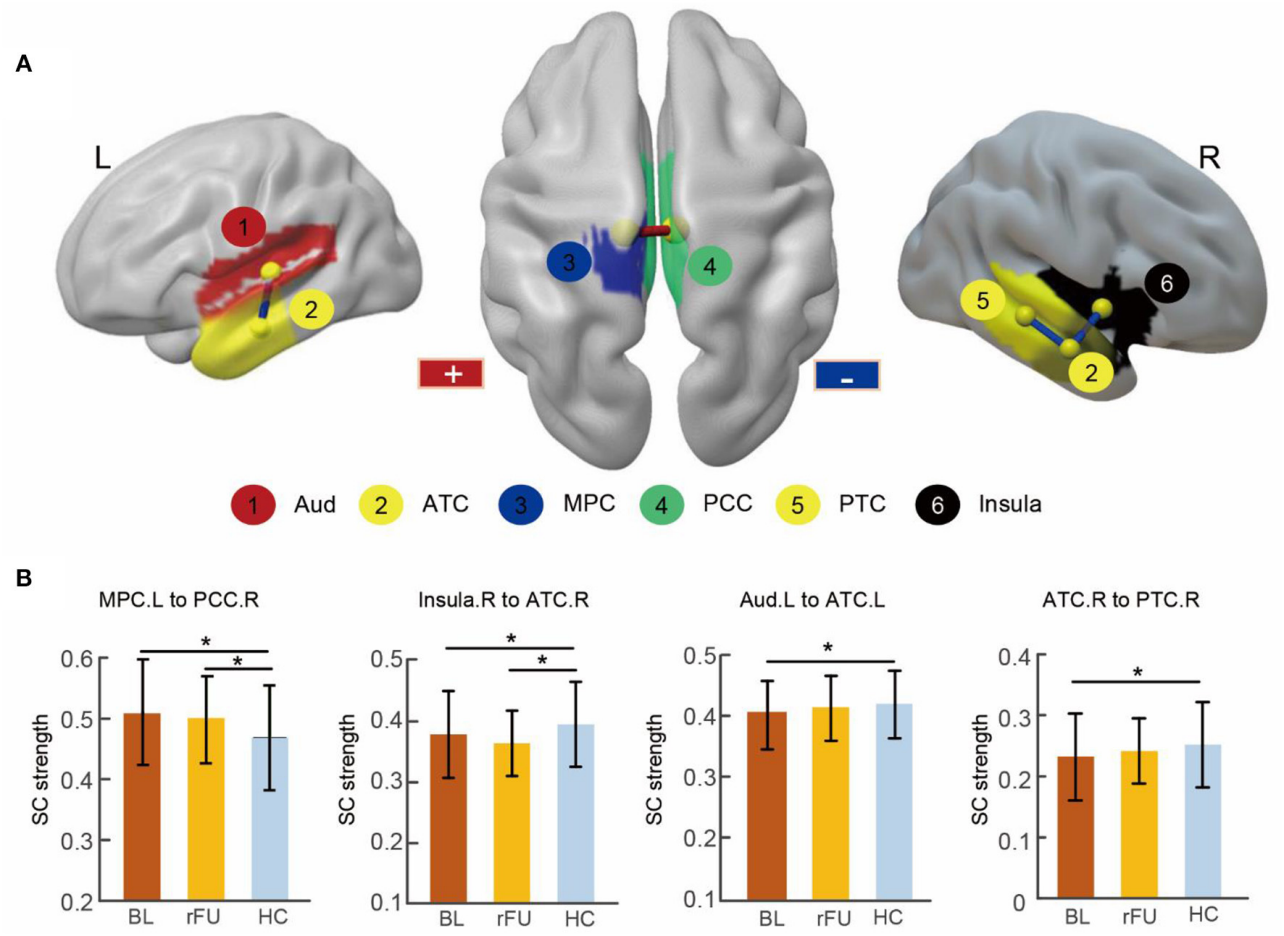
Abnormal SC in MDD patients at baseline and remitted patients at follow-up. **(A)** Three-dimensional representations of the abnormal connectivity in patients at baseline. The regions were mapped onto the cortical surface at a lateral and superior view. The red line represents increased SC in patients at baseline, blue lines represent decreased SC in patients at baseline. **(B)** The bar figures showed the connectivity strengths across the three groups. ^*^ Two-sample *t*-tests (*p* < 0.05, FDR corrected). BL, MDD patients at baseline; rFU, remitted MDD subgroup at follow-up; HC, healthy control group; L, left; R, right; Aud, auditory cortex; ATC, anterior temporal cortex; MPC, medial parietal cortex; PCC, posterior cingulate cortex; PTC, posterior temporal cortex.

Two-sample *t*-tests (*p* < 0.05, FDR corrected) revealed that four connections were significantly different in MDD patients at baseline when compared with HCs, including decreased connectivity between the right insula and the right anterior temporal cortex (ATC), between the right ATC and posterior temporal cortex (PTC), between the left ATC and the auditory cortex as well as increased connectivity between the right posterior cingulate cortex (PCC) and the left medial parietal cortex (MPC). These altered connections were shown in Figure 2 and Table 2.

**Table 2:** Structural connectivity abnormalities in patients at baseline and in remitted patients at follow-up.

**Connectivity**		**Connectivity strength (Mean ± SD)**			***p*****-value**		
**Region 1**	**Region 2**	**BL**	**rFU**	**HC**	^*^BL vs. HC	^*^rFU vs. HC	**^**Δ**^rBL vs. rFU**
Ins.R	ATC.R	0.37 ± 0.07	0.36 ± 0.06	0.39 ± 0.07	0.044	0.004	0.105
PCC.R	MPC. L	0.51 ± 0.08	0.50 ± 0.07	0.47 ± 0.09	<0.001	0.023	0.171
ATC.R	PTC.R	0.23 ± 0.06	0.24 ± 0.06	0.25 ± 0.07	0.022	0.336	0.348
ATC.L	Aud.L	0.40 ± 0.06	0.42 ± 0.05	0.42 ± 0.06	0.014	0.489	0.112

* *Two-sample t-tests*,

Δ *Paired t-tests (p < 0.05, FDR-correction)*.

*BL, the MDD patients at baseline; rFU, remitted MDD subgroup at follow-up; rBL, remitted MDD subgroup at baseline; HC, healthy control group; vs., versus; SD, standard deviation; L, Left; R, right; ATC, Anterior temporal cortex; Aud, Auditory cortex; MPC, medial parietal cortex; PTC, posterior temporal cortex; Ins, Insula; PCC, posterior Cingulate cortex*.

### SC Abnormalities in the Remitted MDD Subgroup at Follow-Up

There was no significant difference between patients who finished the 6-month follow-up procedure and the drop-outs at baseline (*p* < 0.05, FDR corrected).

After a 6-month treatment, two-sample *t*-tests conducted between remitted MDD subgroup at follow-up and HC group revealed decreased connectivity between the right insula and the right ATC and increased connectivity between the right PCC and the left MPC in remitted patients (*p* < 0.05, FDR corrected; Table 2).

### Changes of SC Abnormalities in the Remitted and Unremitted MDD Subgroups Before and After Treatment

Paired *t*-tests revealed that there was no significant difference before and after treatment in remitter or non-remitters (*p* < 0.05, FDR corrected; Table 2).

### Correlations

There was no significant correlation between HAM-D_24_ scores and four abnormal SCs in the MDD group at baseline (Table 3).

**Table 3:** Association between the HAM-D_24_ scores and four abnormal SCs in patients with MDD at baseline.

**Connectivity**		***r***	***p*-value**
**Region 1**	**Region 2**		
Ins.R	ATC.R	0.03	0.77
PCC.R	MPC. L	0.08	0.32
ATC.R	PTC.R	−0.09	0.3
ATC.L	Aud.L	−0.12	0.17

*L, Left; R, right; ATC, Anterior temporal cortex; Aud, Auditory cortex; MPC, medial parietal cortex; PTC, posterior temporal cortex; Ins, Insula; PCC, posterior Cingulate cortex*.

### Validation Results

The results using AAL parcellation revealed that there was no significant SC alteration in MDD patients at baseline compared with HCs at the whole-brain level. Based on the results using 17-functional network parcellation, we further examined the SC alteration between right insula and the right temporal lobe and between the right PCC and the left PCC and the bilateral precuneus (Supplementary Table 2). Detailed analyses and results were described in the Supplementary Materials. The results were in line with the results obtained from 17-functional network parcellation.

The results using the normalized number of fibers as the weight of the network edge revealed that there was no significant SC alteration in the MDD patients after treatment, while significant SC alterations were found both in MDD patients at baseline and in the remitted MDD subgroup at follow-up (*p* < 0.05, FDR-corrected, Supplementary Table 3). The results were comparable with those of the FA-weighted network analyses (Details of the results were described in the Supplementary Materials).

## Discussion

This longitudinal work examined SC changes during 6 months of antidepressant treatment in a large sample of MDD patients. We found four abnormal structural connections in depressed individuals at baseline as compared to HCs, which mainly distributed in the bilateral temporal lobe, the right insula, the left MPC, the PCC. After 6-month antidepressant treatment, decreased connectivity between the right insula and the right ATC, and increased connectivity between the right PCC and the left MPC persisted in remitted patients, showing a state-independent character. In contrast, the decreased connectivity between the right ATC and the PTC and between the left temporal cortex and the auditory cortex were reversed at remission, showing a state-dependent character.

The right insula usually co-activates with limbic cortices (temporal pole, amygdala), which are associated with social cognition, attention (28). A previous task-related study reported the activation of the anterior insula during processing emotional stimuli, particularly negative stimuli (29). Similarly, another task-related study reported abnormal activation of the temporal lobe during emotional processing tasks in patients with MDD compared to HCs (30). Furthermore, the presence of attentional biases to negative stimuli in MDD was reported (31). Besides, both current and remitted MDD patients selectively attended to and remembered sad faces with happy faces filtered out, when presented happy or sad faces paired with emotionally neutral faces in a dot-probe task (32), suggesting a state-independent character of attentional bias in MDD. SC reduction of the insula in acute MDD, even in remitted MDD, had been reported in previous studies (3–6). Also, FA reduction in insula has been reported in our earlier study in patients with MDD from the acute episode to remission and was regarded as a state-independent character (33). In the present study, we found that decreased SC of the insula retained from the episode phrase to the remission in MDD patients after 6-month treatment, which provides ampler evidence for the state-independent character of insula. Moreover, no correlation was found between the SC changes of the right insula to the right ATC and the HAM-D_24_ scores in the MDD group at baseline. The result indicates that alterations of the SC values are free of clinical status, further implying decreased SC between the right insula and the right ATC is a state-independent character for MDD. Taking this imaging evidence and state-independent character of attentional bias together, we speculate that the sustained decreased structural connection between the right insula and the anterior temporal lobe might underlie the neural basis of negative attentional biases in MDD patients. However, more task-related MRI studies of emotional attention bias are needed in the future to elucidate this speculation. Additionally, clinically remitted MDD patients with decreased insula-related SC by MRI scans are likely to be recurrent and require longer antidepressant treatment (34). Besides, the previous study had suggested that the activation of insula can be increased by transcutaneous vagus nerve stimulation (tVNS), so it may be a future direction to develop physical therapy (e.g., tVNS) for insula or its related brain regions (35).

The PCC connected to intrinsic control networks extensively. Increased activity in PCC had been observed in many cases of internally directed attention, such as episodic memory retrieval, daydreaming, and planning (36). Liberg et al. found that the PCC and the posterior MPC were involved in pre-executive motor production during a motor imagery task (37). Pre-executive motor production is a part of the production of movement, including selection, planning, and preparation (38). Motor imagery, rather than performing it openly, is the psychological rehearsal of a specific action. Prior investigations have reported that motor imagery is functionally equivalent to the pre-executive stages of explicit motion (39–41). Liberg et al. further found that the activities in the PCC and the posterior MPC were altered during the pre-executive stages of motor generation in bipolar depression showing psychomotor retardation. Psychomotor retardation is an essential dimension of symptoms in depression. It is manifested as a general slowdown in movement, sagging posture, reduced facial expressions, slower speed, and lowered speech tone. Persisting psychomotor retardation in the remission phase of MDD has been reported, which is independent of the clinical status (42, 43). Moreover, no significant correlation was found between the SC changes of the right PCC to the left MPC and the HAM-D_24_ scores in the MDD group at baseline. The result indicates that alterations of the SC values are also free of clinical status, further implying increased SC between the right PCC and the left MPC is a state-independent character for MDD. Thus, it is reasonable to deduce that the abnormal SC between the right PCC and the left MPC in remitted MDD may contribute to the residual psychomotor retardation symptoms in the remission phase through mediating the pre-executive motor production.

A previous DTI study found that patients with psychosis showed FA reduction affecting fronto-limbic white matter and associative, projective, and commissural fasciculi in the acute phase and showed FA increase over time after symptom remission (44). Another DTI study found that patients with bipolar I disorder (BD-I) showed reduced FA in the right superior, and inferior longitudinal fasciculi and inferior fronto-occipital relative to HCs and remitted BD-I patients, while remitted BD-I patients are not different from HCs in FA (45). The alterations of microstructural white matter from the acute phase to remission were regarded as state-dependent. In the present study, we found that the reduced connections between the right ATC and the PTC and between the left ATC and the auditory cortex were reversed at remission during a 6-month treatment, showing a state-dependent character. Both structural and functional abnormalities in the temporal lobe show high discriminative power in distinguishing MDD patients from HCs (12). Besides, decreased nodal efficiency and thinner cortical gray matter in the temporal lobe have been found in MDD compared with HCs in cross-sectional studies (10, 46). Whereas, thicker cortical thickness in the temporal cortex in remitters than in non-remitters (47), and normalization of the temporal cortex activity with treatment (48) were found. Also, higher FA was detected in the temporal (superior, middle, and fusiform) regions in remitters relative to the non-remitters (49). We found that the reduced SCs of temporal regions are reversed over time after clinical remission in the study, which provides direct evidence for the state-dependent character of temporal regions. Moreover, the auditory cortex is part of the temporal lobe, processing auditory information in human beings. The temporal lobe, together with other regions like the amygdala, played a significant role in emotional processing as well as social cognition (50), which is a well-established region that underpins the pathophysiology of depression (51). Thus, the abnormality of SC between the left ATC and the left auditory cortex, between the right ATC and the PTC may underlie the emotional symptoms in MDD, which seem to be state-dependent. Notably, there was no significant correlation between temporal-related SC alterations and HAMD_24_ scores. Combined with the results that no significant SC alterations were found in the MDD patients after treatment, we are not difficult to infer that the reversion of abnormal SCs is slower than that of depressive symptoms.

This study has a relatively large sample size. However, several limitations should be noted. First, due to some unavoidable reasons (such as migrant workers, severe gastrointestinal reactions, etc.), we lost contact with some of the MDD patients in the follow-up. But patients who finished the 6-month follow-up procedure represent the patients in the entire MDD group at baseline. Thus, the drop-outs at follow-up would not significantly influence our main results. Second, we just collected the MRI scans at two-time points, so we are unable to know more detailed information about the trajectory of SC changes from acute depression to remission. Third, the depressed patients were followed for only 6 months, so it is unable to collect the information of recurrence in the broader period. We are unable to examine whether these state-independent SC abnormalities are associated with a higher risk of recurrence. Fourth, only nine patients did not remit after 6-month treatment during follow-up. Due to the insufficient non-remitters, analyses of this subgroup are exploratory. Fifth, we only analyzed the DTI data, and the results were not able to explain more abnormality of brain functioning. And the subjects were interviewed before and 6 months after treatment, and patients experienced much more during 6 months between two interviews, which we did not know may influence the results of the study.

With a relatively large sample to date, this longitudinal study investigated the state-independent and -dependent SC alterations in patients with MDD. The results demonstrated that SC abnormalities distributed in the right insula, the left PCC, and the right MPC showed a state-independent character, which may be implicated in the sustained negative attention bias and motor retardation in MDD, In contrast, SC abnormalities within the bilateral temporal lobes showed a state-dependent character, which may be associated with the fluctuating affective symptoms in MDD. To investigate the state-independent and state-dependent SCs across the course of depression thoroughly, longitudinal studies with longer follow-up time and more intensive time points will be needed in the future.

## Data Availability Statement

The original contributions presented in the study are included in the article/Supplementary Material, further inquiries can be directed to the corresponding author/s.

## Ethics Statement

The studies involving human participants were reviewed and approved by the Second Xiangya Hospital of Central South University and Zhumadian Psychiatric Hospital. The patients/participants provided their written informed consent to participate in this study.

## Author Contributions

YF analyzed the data, wrote, submitted, and revised the manuscript. QD, XL, JSun, LZ, and MW collected data. JSu, LP, HS, JR, JL, and BL revised the manuscript. L-LZ, DH, and LL conceptualized the study. All authors contributed to the article and approved the submitted version.

## Conflict of Interest

The authors declare that the research was conducted in the absence of any commercial or financial relationships that could be construed as a potential conflict of interest.
