# Supplementary material for: State-Independent and -Dependent Structural Connectivity Alterations in Depression
Source: Front Psychiatry. 2020 Nov 30;11:568717. doi: 10.3389/fpsyt.2020.568717 (PMC7733996; doi:10.3389/fpsyt.2020.568717)
Supplement: Supplementary file 1 [file Data_Sheet_1.PDF]

Supplementary Materials

The results using AAL parcellation revealed that there was no significant SC alteration in MDD patients at baseline compared with HCs at the whole-brain level. Based on the results using 17-functional network parcellation, we further examined the SC alteration between right insula and the right temporal lobe (including regions of Superior_Temporal_R, Superior_Temporal_Pole_R, Middle_Temporal_R, Middle_Temporal_Pole_R). The results revealed that there was a slight SC decrease between the right insula and the Middle_Temporal_R in the MDD patients at baseline compared with HCs (Table S2). After a 6-month treatment, the slightly decreased SC was even lower in the remitted MDD subgroup at follow-up instead of improving. The results were in line with the results obtained from 17-functional network parcellation. Besides, we further examined the SC alteration between the right PCC and the bilateral precuneus as well as the left PCC (SCs of the Cingulum_Post_R to the Cingulum_Post_L, the Cingulum_Post_R to the left precuneus, and the Cingulum_Post_R to the right precuneus). The results revealed that there was a slight SC increase between the right PCC and the left PCC as well as the left precuneus in the MDD patients at baseline compared with HCs (Table S2). After a 6-month treatment, the two slightly increased SCs were even higher in the remitted MDD subgroup at follow-up. The results were also consistent with the results obtained from 17-functional network parcellation.

The results using the number of fibers as the weight of the network edge revealed that there was no significant SC alteration in the MDD patients after treatment, while significant SC alterations were found both in MDD patients at baseline and in the remitted MDD subgroup at follow-up ($p < 0.05$, FDR-corrected). The results revealed that there were three decreased SCs (between the left insula and the left temporo-parietal cortex, between the left dorsal prefrontal cortex and the right medial prefrontal cortex, and the left medial prefrontal cortex and the left orbitofrontal cortex) and an increased SC (between the left medial parietal cortex and the right medial parietal cortex) in the MDD patients at baseline compared with HCs (Table S3). After a 6-month treatment, the decreased SCs and increased SC persisted in the remitted MDD subgroup at follow-up. The results are presented in Table S3. The results were comparable with those of the FA-weighted network analyses.

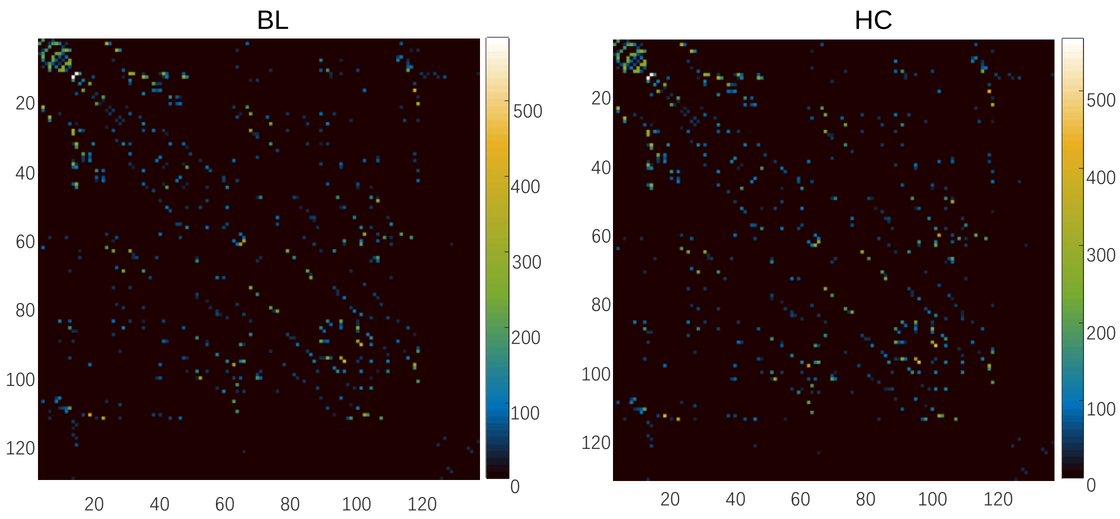

Figure S1. An overview of the average number of fibers tracts between different nodes in MDD patients at baseline and in HCs. Three hundred and seven-eight SC in MDD patients at baseline and 354 SC in HCs survived after one-sample t-test, respectively ($p < 0.05$, FDR corrected). The list of brain regions of the network is shown in Table S1. The color bar displays the average number of fiber tracts. BL = MDD patients at baseline, HC = healthy control group.

Table S1 the list of 132 brain regions of the functional parcellation.

Label	Region names (abbreviation)	Region names (full name)	MNI coordinates	Network
1	Striate.L	Striate cortex	(-9,-97,-6)	VisCent
2	ExStr.L	Extrastriate cortex	(-29,-83,0)	VisCent
3	Striate.L	Striate cortex	(10,-97,-1)	VisCent
4	ExStr.R	Extrastriate cortex	(31,-80,-1)	VisCent
5	Striate.L	Striate cortex	(-10,-76,8)	VisPeri
6	ExStrInf.L	Extrastriate cortex, inferior division	(-15,-58,-3)	VisPeri
7	ExStrSup.L	Extrastriate cortex, superior division	(-10,-79,27)	VisPeri
8	Striate.R	Striate cortex	(12,-73,9)	VisPeri
9	ExStrInf.R	Extrastriate cortex, inferior division	(16,-56,-3)	VisPeri
10	ExStrSup.R	Extrastriate cortex, superior division	(12,-76,29)	VisPeri
11	SomMotA.L	Somatomotor cortex	(-23,-23,63)	SomMotA
12	SomMotA.R	Somatomotor cortex	(22,-20,63)	SomMotA
13	Cent.L	Central sulcus	(-52,-7,35)	SomMotB
14	S2. L	Secondary somatosensory cortex	(-50,-15,17)	SomMotB
15	Ins.R	Insula	(-34,-21,10)	SomMotB
16	Aud.L	Auditory cortex	(-51,-19,7)	SomMotB
17	Cent.R	Central sulcus	(54,-3,33)	SomMotB
18	S2. R	Secondary somatosensory cortex	(48,-10,16)	SomMotB
19	Ins.R	Insula	(36,-18,9)	SomMotB
20	Aud.R	Auditory cortex	(55,-12,6)	SomMotB
21	TOC.L	Temporo-occipital cortex	(-45,-59,-8)	DorsAttnA

Table S1 (continued)

Label	Region names (abbreviation)	Region names (full name)	MNI coordinates	Network
22	POC.L	Parieto-occipital cortex	(-41,-77,24)	DorsAttnA
23	SPL.L	Superior parietal lobule	(-22,-66,53)	DorsAttnA
24	TOC.R	Temporo-occipital cortex	(49,-52,-11)	DorsAttnA
25	POC.R	Parieto-occipital cortex	(46,-70,20)	DorsAttnA
26	SPL.R	Superior parietal lobule	(25,-62,55)	DorsAttnA
27	TOC.L	Temporo-occipital cortex	(-54,-62,10)	DorsAttnB
28	PostC.L	Post-central cortex	(-36,-38,55)	DorsAttnB
29	FEF.L	Frontal eye fields	(-28,-2,55)	DorsAttnB
30	PrCv.L	Ventral pre-central cortex	(-53,8,37)	DorsAttnB
31	TOC.R	Temporo-occipital cortex	(58,-58,6)	DorsAttnB
32	PostC.R	Post-central cortex	(34,-34,55)	DorsAttnB
33	FEF.R	Frontal eye fields	(27,-1,57)	DorsAttnB
34	PrCv.R	Ventral pre-central cortex	(54,12,30)	DorsAttnB
35	ParOper.L	Parietal operculum	(-59,-29,27)	SalVentAttnA
36	PrCv.L	Ventral pre-central cortex	(-51,7,7)	SalVentAttnA
37	Ins.L	Insula	(-39,-2,-2)	SalVentAttnA
38	MPC.L	Medial parietal cortex	(-12,-31,46)	SalVentAttnA
39	MPF.L	Medial frontal cortex	(-7,6,50)	SalVentAttnA
40	ParOper.R	Parietal operculum	(60,-25,28)	SalVentAttnA
41	PrC.R	Pre-central cortex	(50,4,49)	SalVentAttnA
42	PrCv.R	Ventral pre-central cortex	(51,9,6)	SalVentAttnA
43	Ins.R	Insula	(40,1,-3)	SalVentAttnA
44	MPC.R	Medial parietal cortex	(12,-30,47)	SalVentAttnA

Table S1 (continued)

Label	Region names (abbreviation)	Region names (full name)	MNI coordinates	Network
45	MFC.R	Medial frontal cortex	(7,9,49)	SalVentAttnA
46	IPL.L	Inferior parietal lobule	(-61,-40,36)	SalVentAttnB
47	PFCd.L	Dorsal prefrontal cortex	(-15,11,65)	SalVentAttnB
48	PFCl.L	Lateral prefrontal cortex	(-32,46,25)	SalVentAttnB
49	PFCv.L	Ventral prefrontal cortex	(-35,22,-1)	SalVentAttnB
50	OFC.L	Orbitofrontal cortex	(-29,41,-12)	SalVentAttnB
51	PFCmp.L	Posterior-medial prefrontal cortex	(-6,25,33)	SalVentAttnB
52	IPL.R	Inferior parietal lobule	(62,-35,38)	SalVentAttnB
53	PFCd.R	Dorsal prefrontal cortex	(13,15,65)	SalVentAttnB
54	PFCl.R	Lateral prefrontal cortex	(32,49,25)	SalVentAttnB
55	PFClv.R	Ventrolateral prefrontal cortex	(45,46,0)	SalVentAttnB
56	PFCv.R	Ventral prefrontal cortex	(40,24,0)	SalVentAttnB
57	PFCmp.R	Posterior-medial prefrontal cortex	(6,28,33)	SalVentAttnB
58	Cinga.R	anterior cingulate cortex	(5,19,23)	SalVentAttnB
59	TempPole.L	Temporal pole	(-37,-5,-33)	LimbicA
60	TempPole.R	Temporal pole	(37,-3,-34)	LimbicA
61	OFC.L	Orbitofrontal cortex	(-13,37,-19)	LimbicA
62	OFC.L	Orbitofrontal cortex	(12,36,-20)	LimbicA
63	PTC.L	posterior temporal cortex	(-56,-56,-12)	ContA
64	IPS.L	Intraparietal sulcus	(-39,-50,47)	ContA
65	PFCd.L	Dorsal prefrontal cortex	(-24,11,54)	ContA
66	PFCl.L	Lateral prefrontal cortex	(-43,16,28)	ContA
67	PFClv.L	Ventrolateral prefrontal cortex	(-43,39,11)	ContA

Table S1 (continued)

Label	Region names (abbreviation)	Region names (full name)	MNI coordinates	Network
68	Cinga.L	anterior cingulate cortex	(-4,6,28)	ContA
69	PTC.R	posterior temporal cortex	(61,-48,-8)	ContA
70	IPS.R	Intraparietal sulcus	(41,-48,48)	ContA
71	PFCd.R	Dorsal prefrontal cortex	(25,13,53)	ContA
72	PFCl.R	Lateral prefrontal cortex	(45,24,22)	ContA
73	Cinga.R	anterior cingulate cortex	(6,6,29)	ContA
74	PTC.L	posterior temporal cortex	(-60,-40,-14)	ContB
75	IPL.L	Inferior parietal lobule	(-48,-52,52)	ContB
76	PFCd.L	Dorsal prefrontal cortex	(-27,19,59)	ContB
77	PFCl.L	Lateral prefrontal cortex	(-43,26,39)	ContB
78	PFClv.L	Ventrolateral prefrontal cortex	(-34,56,-3)	ContB
79	PFCmp.L	Posterior-medial prefrontal cortex	(-5,32,44)	ContB
80	PTC.R	posterior temporal cortex	(63,-29,-16)	ContB
81	IPL.R	Inferior parietal lobule	(52,-49,49)	ContB
82	PFCld.R	Dorsolateral prefrontal cortex	(39,24,46)	ContB
83	PFClv.R	Ventrolateral prefrontal cortex	(32,55,-4)	ContB
84	PFCmp.R	Posterior-medial prefrontal cortex	(4,39,42)	ContB
85	pCun.L	Precuneus	(-7,-64,46)	ContC
86	PCC.L	Posterior cingulate cortex	(-6,-25,29)	ContC
87	pCun.R	Precuneus	(8,-61,45)	ContC
88	PCC.R	Posterior cingulate cortex	(6,-24,29)	ContC
89	IPL.L	Inferior parietal lobule	(-45,-67,39)	DefaultA
90	PFCd.L	Dorsal prefrontal cortex	(-23,32,44)	DefaultA

Table S1 (continued)

Label	Region names (abbreviation)	Region names (full name)	MNI coordinates	Network
91	PCC.L	Posterior cingulate cortex	(-6,-50,33)	DefaultA
92	PFCm.L	Medial prefrontal cortex	(-9,51,3)	DefaultA
93	PTC.R	posterior temporal cortex	(62,-5,-17)	DefaultA
94	IPL.R	Inferior parietal lobule	(51,-56,32)	DefaultA
95	PFCd.R	Dorsal prefrontal cortex	(23,39,41)	DefaultA
96	PCC.R	Posterior cingulate cortex	(7,-50,32)	DefaultA
97	PFCm.R	Medial prefrontal cortex	(7,49,4)	DefaultA
98	PTC.L	posterior temporal cortex	(-57,-12,-18)	DefaultB
99	IPL.L	Inferior parietal lobule	(-53,-54,30)	DefaultB
100	PFCd.L	Dorsal prefrontal cortex	(-10,47,39)	DefaultB
101	PFCl.L	Lateral prefrontal cortex	(-40,17,49)	DefaultB
102	PFCv.L	Ventral prefrontal cortex	(-46,28,-3)	DefaultB
103	PTC.R	posterior temporal cortex	(63,-25,-4)	DefaultB
104	ATC.R	Anterior temporal cortex	(51,5,-30)	DefaultB
105	PFCd.R	Dorsal prefrontal cortex	(9,50,39)	DefaultB
106	PFCv.R	Ventral prefrontal cortex	(46,29,-8)	DefaultB
107	IPL.L	Inferior parietal lobule	(-43,-77,32)	DefaultC
108	Rsp.L	Retrosplenial cortex	(-12,-57,15)	DefaultC
109	PHC.L	Parahippocampal cortex	(-27,-31,-18)	DefaultC
110	IPL.R	Inferior parietal lobule	(49,-70,30)	DefaultC
111	Rsp.R	Retrosplenial cortex	(14,-53,16)	DefaultC
112	PHC.R	Parahippocampal cortex	(27,-28,-19)	DefaultC
113	TempPar.L	Temporo-parietal cortex	(-58,-33,7)	DefaultD

Table S1 (continued)

Label	Region names (abbreviation)	Region names (full name)	MNI coordinates	Network
114	TempPar.R	Temporo-parietal cortex	(56,-28,4)	DefaultD
115	Putamen.L	Putamen	(-30,-9,6)	SomMotA
116	Putamen.R	Putamen	(31,-6,5)	SomMotA
117	Putamen.L	Putamen	(-28,-10,-2)	SomMotB
118	Putamen.R	Putamen	(29,-7,-3)	SomMotB
119	Putamen.L	Putamen	(-27,-10,7)	SalVentAttnA
120	Putamen.R	Putamen	(28,-6,7)	SalVentAttnA
121	Putamen.L	Putamen	(-24,6,1)	SalVentAttnB
122	Putamen.R	Caudate	(25,7,-1)	SalVentAttnB
123	Caudate.L	Caudate	(-12,11,-8)	LimbicB
124	Caudate.R	Caudate	(12,13,-7)	LimbicB
125	Putamen.L	Putamen	(-30,-15,-5)	ContB
126	Putamen.R	Putamen	(31,-14,-5)	ContB
127	Caudate.L	Caudate	(-13,5,15)	ContC
128	Caudate.R	Caudate	(14,8,15)	ContC
129	Caudate.L	Caudate	(-12,14,0)	DefaultC
130	Caudate.R	Caudate	(12,16,0)	DefaultC
131	Caudate.L	Caudate	(-13,12,15)	DefaultD
132	Putamen.L	Putamen	(-28,-2,-2)	DefaultD

control components A, B, and C (ContA, ContB, ContC); default mode components A, B, C, and D (DefaultA, DefaultB, DefaultC, DefaultD); dorsal attention components A, B (DorsAttnA, DorsAttnB); limbic components A, B (LimbicA, LimbicB); saliency/ventral attention components A, B (SalVentAttnA, SalVentAttnB); somatomotor components A, B (SomMotA, SomMotB); visual central (VisCent) and peripheral (VisPeri) components.

Table S2. SC alterations in MDD patients at baseline and in the remitted MDD subgroup at follow-up based on AAL parcellation.

Connectivity		Connectivity strength (Mean±SD)			*p-value*		
Region 1	Region 2	BL	rFU	HC	*BL vs. HC	*rFU vs. HC	△rBL vs. rFU
Insula.R	Temporal Pole Mid.R	0.17 ± 0.19	0.13±0.19	0.18±0.22	0.70	0.08	0.68
Cingulum Post.R	Cingulum Post.L	0.36 ± 0.1	0.35±0.09	0.34±0.09	0.10	0.31	0.45
Cingulum Post.R	Precuneus.L	0.39 ± 0.22	0.43±0.16	0.39±0.2	0.78	0.22	0.56

*Two-sample *t*-tests, △Paired *t*-tests ($p < 0.05$, FDR-correction).

BL, the MDD patients at baseline; rFU, remitted MDD subgroup at follow-up; rBL, remitted MDD subgroup at baseline; HC, healthy control group; vs., versus; SD, standard deviation; L, Left; R, right. AAL, Automated Anatomical Labeling

Table S3 Significant SC alterations in MDD patients at baseline and in the remitted MDD subgroup at follow-up using the normalized number of fibers as the weight of the network edge.

Connectivity		Connectivity strength (Mean±SD)			*p-value*		
Region 1	Region 2	BL	rFU	HC	*BL vs. HC	*rFU vs. HC	△rBL vs. rFU
Ins.L	TPC.L	0.0±0.00	0.0±0.00	0.16±0.06	< 0.001	< 0.001	1
MPC.R	MPC. L	0.14±0.08	0.12±0.07	0.0±0.00	< 0.001	< 0.001	0.479
PFCd.L	MFC.R	0.0±0.00	0.0±0.00	0.16±0.12	< 0.001	< 0.001	1
PFCm.L	OFC.R	0.0±0.00	0.0±0.00	0.16±0.08	< 0.001	< 0.001	1

*Two-sample *t*-tests, △Paired *t*-tests ($p < 0.05$, FDR-correction).

BL, the MDD patients at baseline; rFU, remitted MDD subgroup at follow-up; rBL, remitted MDD subgroup at baseline; HC, healthy control group; vs., versus; SD, standard deviation; L, Left; R, right; TPC = Temporo-parietal cortex, MPC = medial parietal cortex, PFCd = Dorsal prefrontal cortex, Ins = Insula, PFCm = Medial prefrontal cortex, OFC = Orbitofrontal cortex.
